# Copeptin as a marker of outcome after cardiac arrest: a sub-study of the TTM trial

**DOI:** 10.1186/s13054-020-02904-8

**Published:** 2020-04-28

**Authors:** Joachim Düring, Martin Annborn, Tobias Cronberg, Josef Dankiewicz, Yvan Devaux, Christian Hassager, Janneke Horn, Jesper Kjaergaard, Michael Kuiper, Homa Rafi Nikoukhah, Pascal Stammet, Johan Undén, Michael Jaeger Wanscher, Matt Wise, Hans Friberg, Niklas Nielsen

**Affiliations:** 1 Department of Clinical Sciences, Anesthesia & Intensive care, Lund University, Skåne University Hospital, Malmö, Sweden; 2 Department of Clinical Sciences Lund, Anesthesia & Intensive care, Lund University, Helsingborg Hospital, Helsingborg, Sweden; 3 Department of Clinical Sciences, Neurology, Lund University, Skåne University Hospital, Lund, Sweden; 4 Department of Clinical Sciences, Cardiology, Lund University, Skåne University Hospital, Lund, Sweden; 5 Cardiovascular Research Unit, Luxembourg Institute of Health, Strassen, Luxembourg; 6 Department of Cardiology, Rigshospitalet and Dept of Clinical Medicine, University of Copenhagen, Copenhagen, Denmark; 7 Department of Intensive Care, Amsterdam UMC, University of Amsterdam, Amsterdam, The Netherlands; 8 Department of Intensive Care, Medical Center Leeuwarden, Leeuwarden, The Netherlands; 9 Thermo Fisher Scientific, Asnières sur Seine, France; 10 Medical and Health Directorate, National Fire and Rescue Corps, 1, rue Stümper, 2557 Luxembourg, Luxembourg; 11 Department of Clinical Sciences Lund, Anesthesia & Intensive care, Lund University, Halmstad Hospital, Halmstad, Sweden; 12 Department of Cardiothorasic anesthesia, Rigshospitalet and Dept of Clinical medicine, University of Copenhagen, Copenhagen, Denmark; 13 Adult Critical Care, University Hospital of Wales, Cardiff, UK

**Keywords:** Humans, Copeptin, Arginine vasopressin, AVP protein human, Out-of hospital cardiac arrest, Biomarkers, Critical illness, Prognosis, Survivors

## Abstract

**Background:**

Arginine vasopressin has complex actions in critically ill patients, involving vasoregulatory status, plasma volume, and cortisol levels. Copeptin, a surrogate marker for arginine vasopressin, has shown promising prognostic features in small observational studies and is used clinically for early rule out of acute coronary syndrome. The objective of this study was to explore the association between early measurements of copeptin, circulatory status, and short-term survival after out-of-hospital cardiac arrest.

**Methods:**

Serial blood samples were collected at 24, 48, and 72 h as part of the target temperature management at 33 °C versus 36 °C after cardiac arrest trial, an international multicenter randomized trial where unconscious survivors after out-of-hospital cardiac arrest were allocated to an intervention of 33 or 36 °C for 24 h. Primary outcome was 30-day survival with secondary endpoints circulatory cause of death and cardiovascular deterioration composite; in addition, we examined the correlation with extended the cardiovascular sequential organ failure assessment (eCvSOFA) score.

**Results:**

Six hundred ninety patients were included in the analyses, of whom 203 (30.3%) developed cardiovascular deterioration within 24 h, and 273 (39.6%) died within 30 days. Copeptin measured at 24 h was found to be independently associated with 30-day survival, hazard ratio 1.17 [1.06–1.28], *p* = 0.001; circulatory cause of death, odds ratio 1.03 [1.01–1.04], *p* = 0.001; and cardiovascular deterioration composite, odds ratio of 1.05 [1.02–1.08], *p* < 0.001. Copeptin at 24 h was correlated with eCvSOFA score with rho 0.19 [0.12–0.27], *p* < 0.001.

**Conclusion:**

Copeptin is an independent marker of severity of the post cardiac arrest syndrome, partially related to circulatory failure.

**Trial registration:**

Clinical Trials, NCT01020916. Registered November 26, 2009.

## Introduction

Hypoxic ischemic encephalopathy (HIE) is the major determinant of outcome after out-of-hospital cardiac arrest (OHCA) [1]. Existing prognostication models are targeted at neurologic functional outcome. Death is biphasic after OHCA with early death (1–3 days) to a large degree related to circulatory failure, while later death (> 3 days) is mainly related to withdrawal of life-sustaining therapy (WLST) due to assumed severe HIE [2]. Patients with a presumed high risk of circulatory-related death may therefore benefit from extended hemodynamic monitoring and support. This is particularly relevant for patients without signs of severe HIE and a potential good long-term outcome. A risk stratification model addressing this topic has recently been presented but did not include the use of biomarkers [3].

Arginine vasopressin (AVP) is a peptide hormone released from the posterior pituitary gland that increases solute-free water reabsorption in the renal tubules and systemic vascular resistance by constricting selected arterioles. Elevated AVP levels have been shown to correlate with shock [4] and cardiovascular failure [5–7]. Measurement of AVP is challenging because of its short half-life, but it can be replaced by measurement of copeptin (also known as CT-proAVP), the C-terminal proteolytic product of the pre-pro-hormone of AVP. Copeptin has shown to be a reliable surrogate biomarker of vasopressin [8], and levels are significantly increased at hospital admission in patients with acute coronary syndrome [9]. Also, high copeptin levels are associated with risk of death in patients with cardiovascular failure [10, 11] while low levels have been implemented in clinical practice to rule out non-ST-segment acute myocardial infarction [9, 12]. Furthermore, copeptin has been suggested as a promising prognostic biomarker after OHCA [13–16]. It is unknown whether the prognostic capabilities of AVP/copeptin are related to cardiovascular failure alone or if copeptin is merely a marker of disease severity. The aim of this exploratory study was to investigate the relationship between early copeptin levels, circulatory failure, and mortality in the setting of OHCA. We hypothesized that (1) early copeptin levels are associated with early mortality and (2) copeptin levels are associated with circulatory failure.

## Materials and methods

### Study design and setting

This is a predefined biomarker sub-study of the target temperature management (TTM) 33 °C versus 36 °C after out-of-hospital cardiac arrest trial [17], randomizing 950 unconscious OHCA patients to an intervention of 33 °C or 36 °C, indicating no significant benefit of a target temperature of 33 °C compared to 36 °C [17]. The trial protocol was approved by ethical committees in each participating country, and informed consent was waived or obtained from all participants or relatives according to national legislation, in line with the Helsinki declaration [18].

### Study population

Unconscious adult patients were included in the TTM trial within 4 h of return of stable spontaneous circulation (ROSC) after OHCA of a presumed cardiac cause. Previous medical history was recorded without no strict definitions. Patient hemodynamics at screening and before randomization were classified in one of three categories: 1, no shock, systolic blood pressure (SBP) > 90 mmHg; 2, moderate shock, SBP < 90 mmHg for > 30 min, or the need of supportive measures (fluid loading, vasopressor, and/or inotropic support) to maintain SBP > 90 mmHg and/or end-organ hypoperfusion (cool extremities, urine output < 30 ml/h); and 3, severe shock, SBP < 80 mmHg in spite of supportive measures (medical and mechanical) that could not be reversed within the inclusion window. Patients in persistent severe shock were not eligible. Of the 36 study sites, seven sites did not participate in the biobank sub-study due to legal or logistical reasons. This analysis included all patients alive at 24 h with at least one recorded copeptin level during the sampling period.

### Sampling and measurements

Blood serum samples were collected at 24, 48, and 72 h after enrolment in the study and were processed at the study sites, aliquoted and frozen to − 80 °C before shipment to the Integrated Biobank of Luxemburg. Centralized batch analysis of copeptin was done using immune fluorescence with the Brahms Kryptor Compact Plus system (Thermo Fisher Scientific Brahms, Germany), with range of 0.7–500 pmol/L (auto dilution with upper range of 2000 pmol/L) and functioning assay sensitivity of 1.08 pmol/L. All serum samples with interference detected by the Kryptor system were discarded. Analyses of copeptin were made at least 6 months after trial completion.

### Outcomes

The primary outcome was incidence of death until 30 days after enrolment in the TTM trial. The secondary endpoints were circulatory cause of death within 30 days of cardiac arrest, as estimated from clinical data, and the extended cardiovascular sequential organ failure assessment score (eCvSOFA), a nine level sub-score of the SOFA cardiovascular component [19]. In addition, we devised a binary composite outcome of cardiovascular deterioration (cardiovascular deterioration composite (CvDC)) considered positive if the patient had an eCvSOFA ≥ 5 or died from circulatory cause at within ± 12 h of copeptin sample or if eCvSOFA score increased more than two points within the previous 24 h.

### Statistical analysis

Descriptive statistics were used to summarize the study population. Continuous data are presented as median with interquartile range. Differences in baseline variables were assessed using the Mann-Whitney or the *χ*^2^ test, as appropriate. Analyses were primarily performed on 24-h samples of copeptin, with the secondary analyses performed on serial samples (48, 72-h samples). No formal adjustment of significance levels due to multitesting was performed. Due to missingness in the dataset, analyses were performed on a pooled dataset for the explanatory models based on 20 imputations by chained equations, using predictive mean matching for continuous variables and logistic regression for categorical data. Because of skewed distribution of copeptin data, log2-transformed copeptin levels were used in Cox and logistic regression models. All models were adjusted for TTM at 33 °C (yes/no) and early clinical predictors of outcome according to the TTM-score [20]: age (years), cardiac arrest at home (yes/no), no-flow time (minutes from cardiac arrest until start of chest compressions or ROSC, whichever was first), low-flow time (minutes with chest compressions), shockable rhythm (yes/no), use of adrenaline during CPR (yes/no), absence of corneal and pupillary reflexes on admission (yes/no), Glasgow coma score motor component (mGCS) on admission > 1 or sedated (yes/no), admission pH, and admission PaCO_2_ < 4.5 kPa (yes/no). The log-rank test was used to test for difference in 30-day survival according to copeptin levels stratified as above or below median at the specified time point in the biobank population and visualized in Kaplan-Meier graphs. Adjusted hazard ratio for death was estimated using the Cox regression of proportional hazards model, with follow-up time censored at 30 days. Multivariate logistic regression was performed for circulatory cause of death. Correlation between copeptin and eCvSOFA-score was assessed using Spearman’s rank order correlation. Multivariate logistic regression was performed for CvDC.

A sensitivity analysis was performed comparing the results of a complete cases adjusted Cox regression model for the primary endpoint at 24, 48, and 72 h with that of the imputed model.

Statistical analysis was performed using software R (v 3.4.0) and RStudio (v 1.0.143).

## Results

A total of 690 patients were included in this TTM biobank sub-study (Table 1). Of 2070 possible copeptin values, 112 (5.4%) were missing, 53 (2.6%) because of death prior to sampling (Additional file 1).

**Table 1:** Baseline characteristics

**Characteristic**	**Patients included in sub-study**
**Subjects**	690
**Temperature intervention at 36 C,*n*** **= 690**	343 (49.7)
**Age, years,*n*** **= 690**	65 [56–73]
**Male sex,*n*** **= 690**	558 (80.9)
**Previous medical history**	
**Myocardial infarction,*n*** **= 684**	134 (19.6)
**Ischemic heart disease,*n*** **= 683**	187 (27.4)
**Arrhythmia,*n*** **= 684**	121 (17.7)
**Arterial hypertension,*n*** **= 682**	277 (40.6)
**Diabetes,*n*** **= 679**	100 (14.7)
**Intra-arrest characteristics**	
**Witnessed cardiac arrest,*n*** **= 687**	614 (89.4)
**Cardiac arrest at home,*n*** **= 690**	364 (52.8)
**Bystander cardiopulmonary resuscitation,*n*** **= 690**	499 (72.3)
**No flow time (min),*n*** **= 679**	1 [0–5]
**Low flow time (min),*n*** **= 679**	22 [14–35]
**Dose of adrenaline (mg),*n*** **= 680**	2 [0–4]
**ST-elevation myocardial infarction,*n*** **= 676**	276 (40.8)
**First monitored rhythm,*n*** **= 690**	
**Non shockable**	128 (18.6)
**Shockable**	542 (78.6)
**Unknown**	20 (2.9)
**Early hospital characteristics**	
**Moderate shock on admission,*n*** **= 686**	85 (12.4)
**Lactate at admission (mmol/l),*n*** **= 619**	5.9 [3.2–9.3]
**Admission pCO**_**2**_**< 4.5 kPa,*n*** **= 645**	80 (11.6)
**Pupillary or corneal reflexes present on admission,*n*** **= 649**	7.23 [7.13–7.31]
**Admission GCSm > 1 or sedated,*n*** **= 679**	308 (44.6)
**Pupillary or corneal reflexes present on admission,*n*** **= 655**	527 (76.4)
**Extended cardiovascular SOFA score day 1,*n*** **= 673**	3 [2–4]
**Positive cardiovascular deterioration composite at 24 h,*n*** **= 670**	203 (30.3%)

Baseline characteristics in the TTM biobank population included in the analysis. Continuous data is presented as median value with interquartile range, while categorical data as number of subjects and percentages. *n* represents the total number of samples available for analysis

### Copeptin and temperature

Differences in copeptin levels between interventions were highest at 72 h, 33.00 [15.76–63.62] for 33 °C and 26.46 [11.37–59.28] pmol/l, for 36 °C, *p* = 0.049 (Additional file 2). Copeptin levels increased more between 24 h and 48 h at an intervention of 33 °C, 6.11 [− 4.25–36.96] compared to 36 °C 1.91 [− 9.51–27.38] pmol/l, (*p* = 0.026), while no significant differences were seen between 48 and 72 h. Since differences between intervention arms were minor and our primary aim was to test associations between copeptin and outcome, all further analyses were performed on pooled samples.

### Copeptin and mortality

**Fig. 1:**
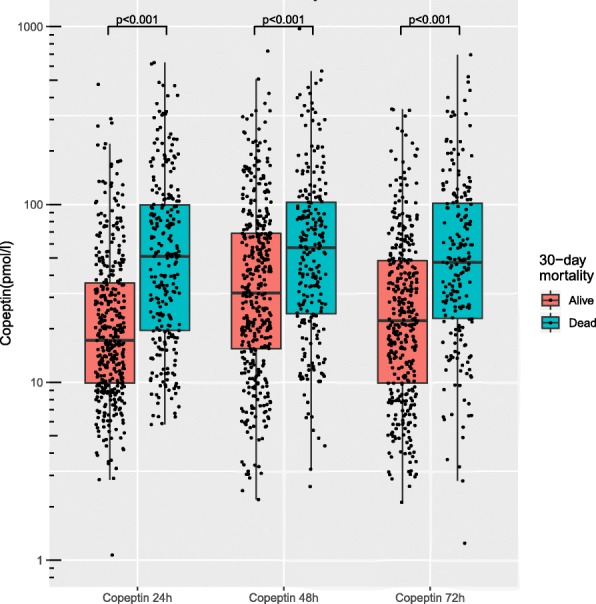
Copeptin levels stratified according to 30-day mortality. Box plot illustrating difference in copeptin levels measured at 24, 48, and 72 h after cardiac arrest in survivors vs non-survivors at day 30. Copeptin on *Y*-axis is on a log scale

**Fig. 2:**
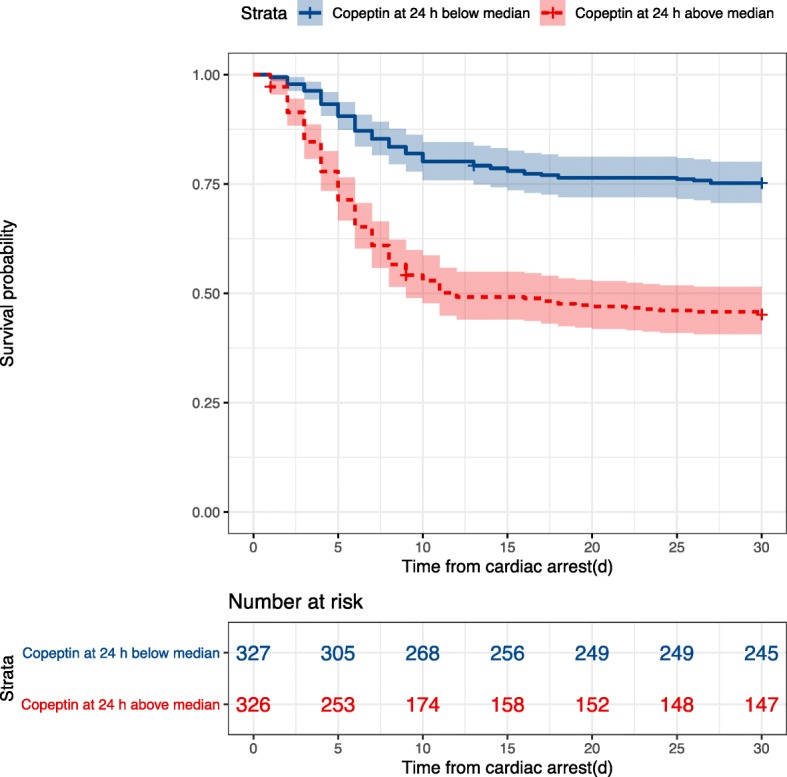
Probability of 30-day survival. Kaplan-Meier graph illustrating the probability of survival after cardiac arrest according to copeptin levels stratified as above or below median at 24 h. Outcome was censored after 30 days. Shaded areas indicate 95% confidence interval. Survival was significantly higher in the group with copeptin levels below median at 24 h, *p* < 0.001

**Fig. 3:**
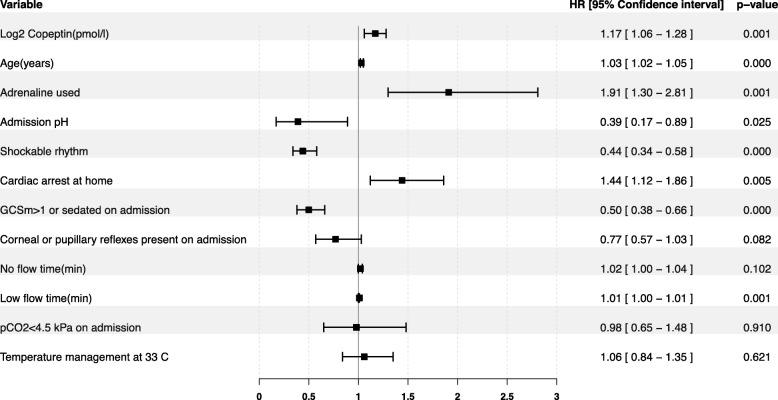
Multivariate explanatory model for short time survival. Forest plot displaying hazard ratio for death within 30 days in a multivariate Cox proportional hazards model adjusted for log2-transformed copeptin at 24 h, age (years), adrenaline used (yes/no), admission pH, shockable rhythm (yes/no), cardiac arrest at home (yes/no), Glasgow Coma Scale motor component (GCSm) more than 1 or sedated at admission (yes/no), corneal or pupillary reflexes present at admission (yes/no), no flow time = time from cardiac arrest until start of chest compression or return of spontaneous circulation, whichever comes first (min), low flow time = from start of chest compressions until return of spontaneous circulation (min), admission arterial pCO2 below 4.5 kPa on admission (yes/no), and temperature management at 33 C after cardiac arrest (yes/no). *p* values below 0.05 were considered significant

Two hundred seventy-three (39.6%) patients died within 30 days. Copeptin levels were significantly lower in survivors than in patients dead by day 30, at 24 h 17.19 [9.89–36.20] versus 51.14 [19.60–99.51] pmol/l, at 48 h 31.84 [15.44–59.49] versus 57.11 [24.32–102.97] pmol/l, and at 72 h 22.19 [9.89–48.26] versus 47.37 [22.90–101.78] pmol/l, all *p* values < 0.001 (Fig. 1). Crude 30-day survival was associated with copeptin stratified as above or below median at 24 h, *p* < 0.001 (Fig. 2), at 48 h, *p* = 0.001, and 72 h *p* < 0.001 (Additional file 3). The incidence of death was independently associated with log2-transformed copeptin, hazard ratio (HR) 1.17 [1.06–1.28], *p* = 0.001(Fig. 3); for samples at 24 h, significance was lost at 48 h, *p* = 0.471, while 72-h samples were trending towards significance, HR 1.11 [1.00–1.23], *p* = 0.054.

### Copeptin and circulatory failure

**Fig. 4:**
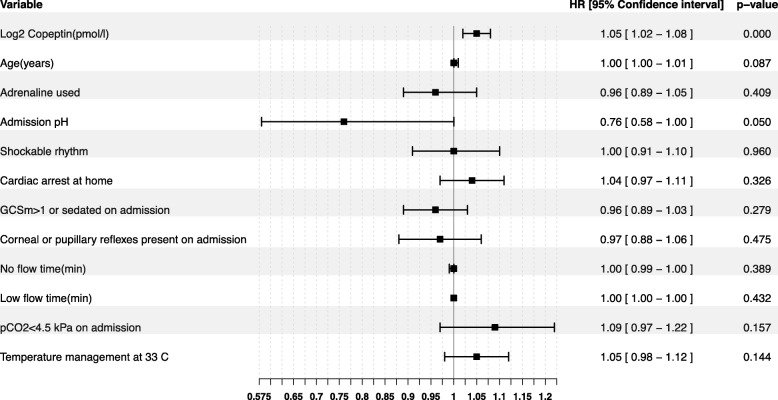
Multivariate explanatory model for cardiovascular deterioration composite, CvDC, at 24 h. Forest plot displaying odds ratios for a positive cardiovascular deterioration composite (CvDC) in a multivariate logistic regression model. CvDC was considered positive if the patient had an extended cardiovascular SOFA score (eCvSOFA) ≥ 5 or died from circulatory cause at within ± 12 h of time point or if eCvSOFA score increased more than two points within the previous 24 h. The model was adjusted for adjusted for log2-transformed copeptin at 24 h, age (years), adrenaline used (yes/no), admission pH, shockable rhythm (yes/no), cardiac arrest at home (yes/no), Glasgow Coma Scale motor component (GCSm) more than 1 or sedated at admission (yes/no), corneal or pupillary reflexes present at admission (yes/no), no flow time = time from cardiac arrest until start of chest compression or return of spontaneous circulation, whichever comes first (min), low flow time = from start of chest compressions until return of spontaneous circulation (min), admission arterial pCO2 below 4.5 kPa on admission (yes/no), and temperature management at 33 °C after cardiac arrest (yes/no). *p* values below 0.05 were considered significant

In the adjusted model, log2-transformed copeptin was independently associated with circulatory cause of death, at 24 h, with an odds ratio (OR) of 1.03 [1.01–1.04], *p* = 0.001 (Additional file 4). The Spearman rank order correlation between eCvSOFA and copeptin, rho, was 0.19 [0.12–0.27] at 24 h, 0.22 [0.14–0.29] at 48 h and 0.26 [0.18–0.34] at 72 h, *p* < 0.001, for all time points. Log2-transformed copeptin was independently associated with cardiovascular deterioration with OR 1.05 [1.02–1.08], *p* < 0.001 at 24 h (Fig. 4); OR 1.03 [1.00–1.05], *p* = 0.021 at 48 h; and OR 1.03 [1.01–1.06], *p* = 0.016 at 72 h.

In the sensitivity analysis, results from the Cox regression models on imputed datasets and observed data were similar (data not shown).

## Discussion

The main findings of this sub-study of the TTM trial were that copeptin is a time sensitive, independent predictor of early mortality after OHCA, weakly associated with cardiovascular failure.

This is to date the largest study investigating copeptin as a marker of outcome after OHCA. Our finding that copeptin was associated with outcome is in agreement with previous studies [13–16, 21, 22]. Studies by Annborn et al. [13, 16] and Broessner et al. [14] indicate better prognostic precision with early measurements of copeptin which could be one reason for copeptin being significantly associated with survival at 24 h only, in our multivariate analysis. Previous studies have indicated a rapid release and clearance of copeptin [23] after circulatory stress, and this may explain why early copeptin levels would be more strongly associated with early mortality. Copeptin was associated with temperature change, and the multivariate analyses are adjusted for TTM, but it cannot be ruled out that the temperature intervention might have confounded the results of the 48 h analysis, since the patients in the intervention group with TTM at 33 °C were rewarmed to 37 °C between 28 and 36 h according to the TTM trial protocol. Death before sampling is a potential confounder that decreased our results for copeptin’s association with death, as these patients probably had high copeptin levels.

As the results of this study indicate an association between early mortality, circulatory status and circulatory cause of death with high early copeptin levels, it seems reasonable to expect that the exclusion of patients with severe hemodynamic shock may have decreased the prognostic precision of copeptin. Since exclusion due to irreversible severe shock in the TTM trial was 2%, we believe this effect, however, to be minor.

Copeptin was independently associated with circulatory endpoints and associated with early mortality in univariate analysis at 24, 48, and 72 h, while copeptin was independently associated with short time survival at 24 h. This might indicate copeptin to represent the composite stress due to a wide array of pathologies, e.g., hypoxia, shock, and infection, rather than to represent circulatory failure alone. This is consistent with copeptin being associated with outcome in various other settings, such as pneumonia, stroke, postoperative trauma, and traumatic brain injury [4, 24–26].

The association of copeptin with CvDC was significant at all time points with modest ORs, which is consistent with the low correlation with eCvSOFA. We did not account for multitesting, but since *p* values were consistently low, we believe copeptin is a true, albeit not clinically relevant, indicator of circulatory status. The independent association of copeptin with circulatory cause of death at 24 h, but not 48 or 72 h, should thus be interpreted cautiously.

The use of copeptin as a clinical screening tool to identify OHCA patients who might benefit from extended hemodynamic monitoring cannot be supported by our results, primarily because copeptin is not associated with circulatory status at any clinically relevant level. Further studies on copeptin are needed to clarify the optimal timing of sampling, the potential value as a marker of circulatory failure or a marker of disease severity in early multivariate prognostic models, including comparisons to other relevant biomarkers (e.g., natriuretic peptides, C-reactive protein, and procalcitonin). Furthermore, more research is warranted to investigate the complex interactions of copeptin as a marker of free water resorption, vasopressor, and cortisol status in the critically injured patient.

### Strengths and limitations

This is a large predefined sub-study of the TTM trial investigating copeptin as a marker of severity of the post cardiac arrest syndrome. The TTM trial had strict rules for withdrawal of life-sustaining therapy, and all clinical data were prospectively collected. Analyses were made in a single laboratory, limiting the risk of inter-laboratory assay variability, and copeptin values were analyzed after trial completion, eliminating the risk of treatment bias. Due to the limitations of an observational study, we can however only assess associations with outcome. It cannot be ruled out that missing data may have affected our results. The collection of blood samples for the biobank of the TTM trial was not specifically designed for this sub-study, and the initial sampling of copeptin at 24 h may have been too late for optimal assessment of associations with outcome and circulatory derangement. Patients dying before 24 h were excluded from our analysis, limiting our results to patients alive at 24 h; also, the exclusion of patients in irreversible hemodynamic shock may limit the generalizability of our results.

## Conclusion

Copeptin is an independent marker of severity of the post cardiac arrest syndrome, partially related to circulatory failure.

## Supplementary information

**Additional file 1.** Flow chart. A, the number of patients enrolled in the TTM-trial and included in the sub-study. B, Illustrates missing copeptin data within 72 h.

**Additional file 2.** Boxplot illustrating difference in copeptin levels measured at 24, 48, and 72 h after cardiac arrest in patients treated with a temperature intervention at 33 C or 36 C. Copeptin on Y-axis is on a log scale. TTM: Targeted Temperature Management.

**Additional file 3. **Kaplan-Meier plots according to copeptin levels stratified as above or below median at 48, and 72 h. Shaded areas indicate 95% confidence interval. Outcome was censored after 30 days. Survival was significantly higher in the group with copeptin levels below median at 48 h, *p* = 0.001 and 72 h, *p* < 0.001.

**Additional file 4. **Forest plot displaying odds ratios for cardiac cause of death within 30 days of cardiac arrest in a multivariate logistic regression mode. The model is adjusted for adjusted for: log2 transformed copeptin at 24 h, age (years), adrenaline used (yes/no), admission pH, Shockable rhythm (yes/no), cardiac arrest at home (yes/no), Glasgow Coma Scale motor component (GCSm) more than 1 or sedated at admission (yes/no), corneal or pupillary reflexes present at admission (yes/no), no flow time = time from cardiac arrest until start of chest compression or return of spontaneous circulation, whichever comes first (min), low flow time = from start of chest compressions until return of spontaneous circulation (min), admission arterial pCO2 below 4.5 kPa on admission (yes/no), and temperature management at 33 C after cardiac arrest (yes/no). *p*-values below 0.05 were considered significant.

## Data Availability

The data that support the findings of this study are available from Niklas Nielsen, but restrictions apply to the availability of these data, which were used under license for the current study, and so are not publicly available. Data are however available from the authors upon reasonable request and with permission of Niklas Nielsen.

## Abbreviations

WLST: Withdrawal of life-sustaining therapy
TTM: Targeted temperature management
SOFA: Systemic organ failure assessment
eCvSOFA: Extended cardiovascular systemic organ failure assessment
HIE: Hypoxic ischemic encephalopathy
OHCA: Out-of-hospital cardiac arrest
AVP: Arginine vasopressin
CT-proAVP: C-terminal pro-arginine vasopressin (copeptin)
ROSC: Return of spontaneous circulation
SBP: Systolic blood pressure
CvDC: Cardiovascular deterioration composite
CPC: Cerebral performance category
mGCS: Motor component of Glasgow Coma Score
STEMI: ST-elevation myocardial infarction
HR: Hazard ratio
OR: Odds ratio
