# Supplementary figures and images for: Copeptin as a marker of outcome after cardiac arrest: a sub-study of the TTM trial
Source: Crit Care. 2020 Apr 28;24:185. doi: 10.1186/s13054-020-02904-8 (PMC7189642; doi:10.1186/s13054-020-02904-8)

A

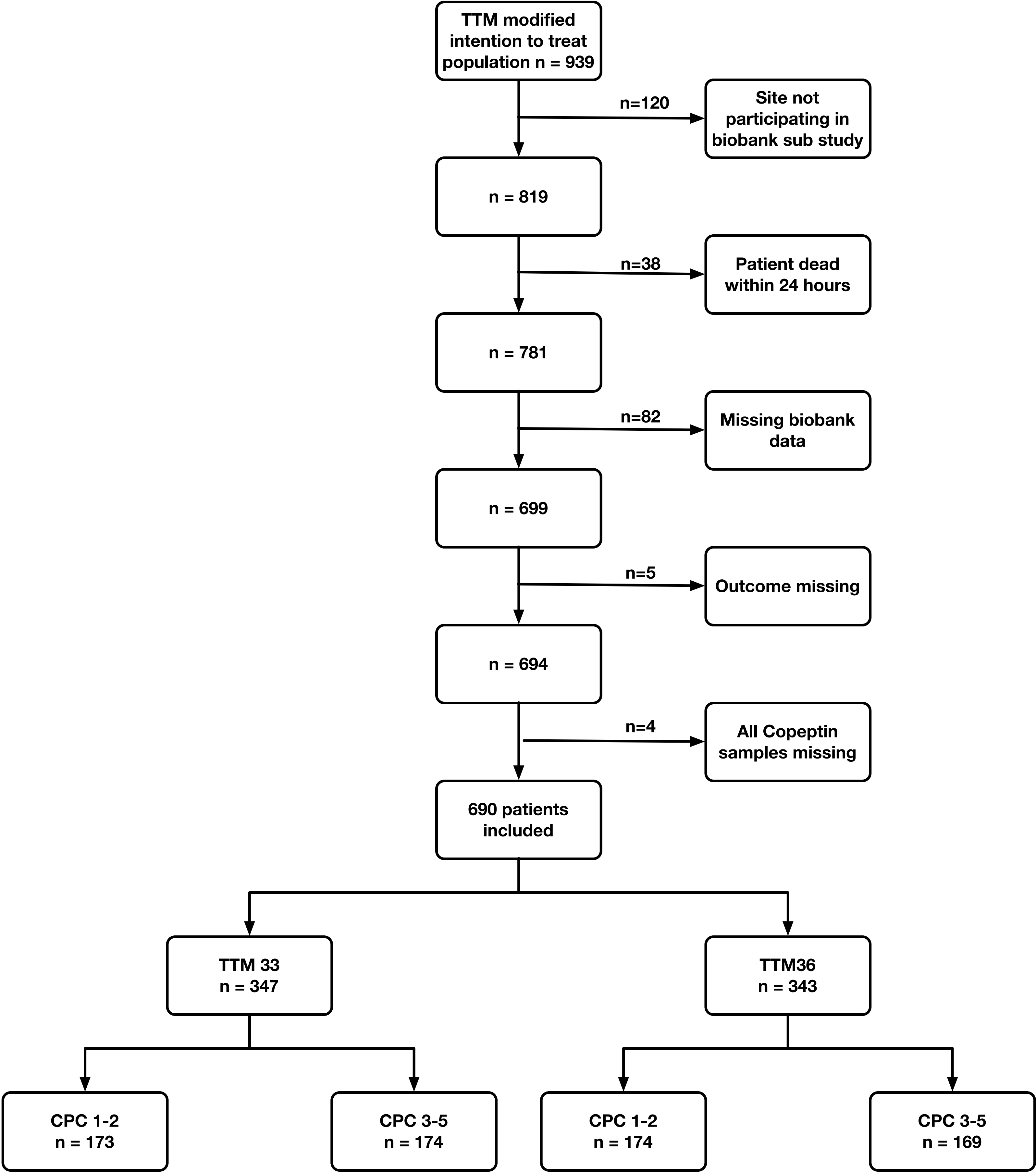

B

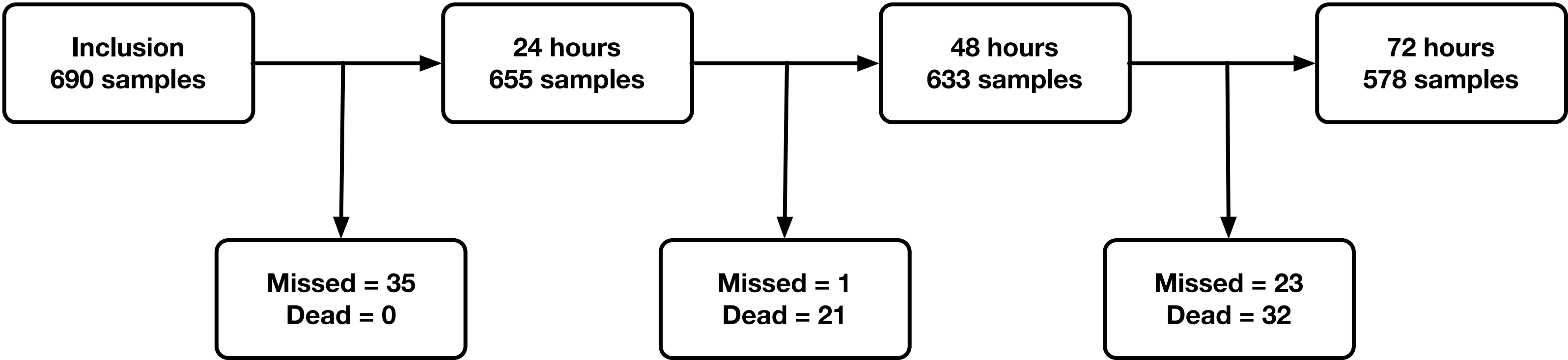

Supplement: Supplementary file 1 — Additional file 1. Flow chart. A, the number of patients enrolled in the TTM-trial and included in the sub-study. B, Illustrates missing copeptin data within 72 h. [file 13054_2020_2904_MOESM1_ESM.pdf]

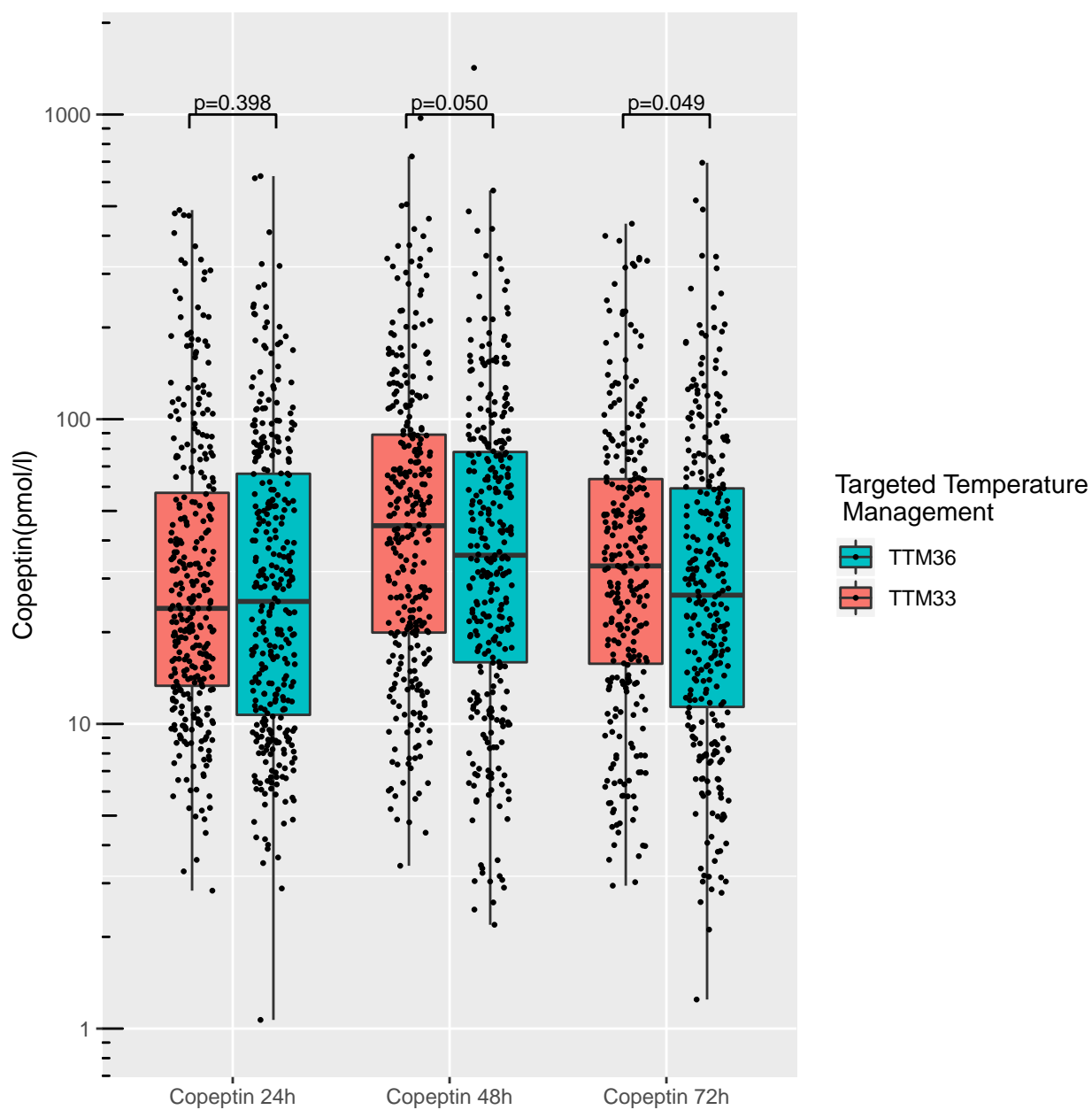

Supplement: Supplementary file 2 — Additional file 2. Boxplot illustrating difference in copeptin levels measured at 24, 48, and 72 h after cardiac arrest in patients treated with a temperature intervention at 33 C or 36 C. Copeptin on Y-axis is on a log scale. TTM: Targeted Temperature Management. [file 13054_2020_2904_MOESM2_ESM.pdf]

Strata 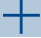 Copeptin below median 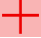 Copeptin above median

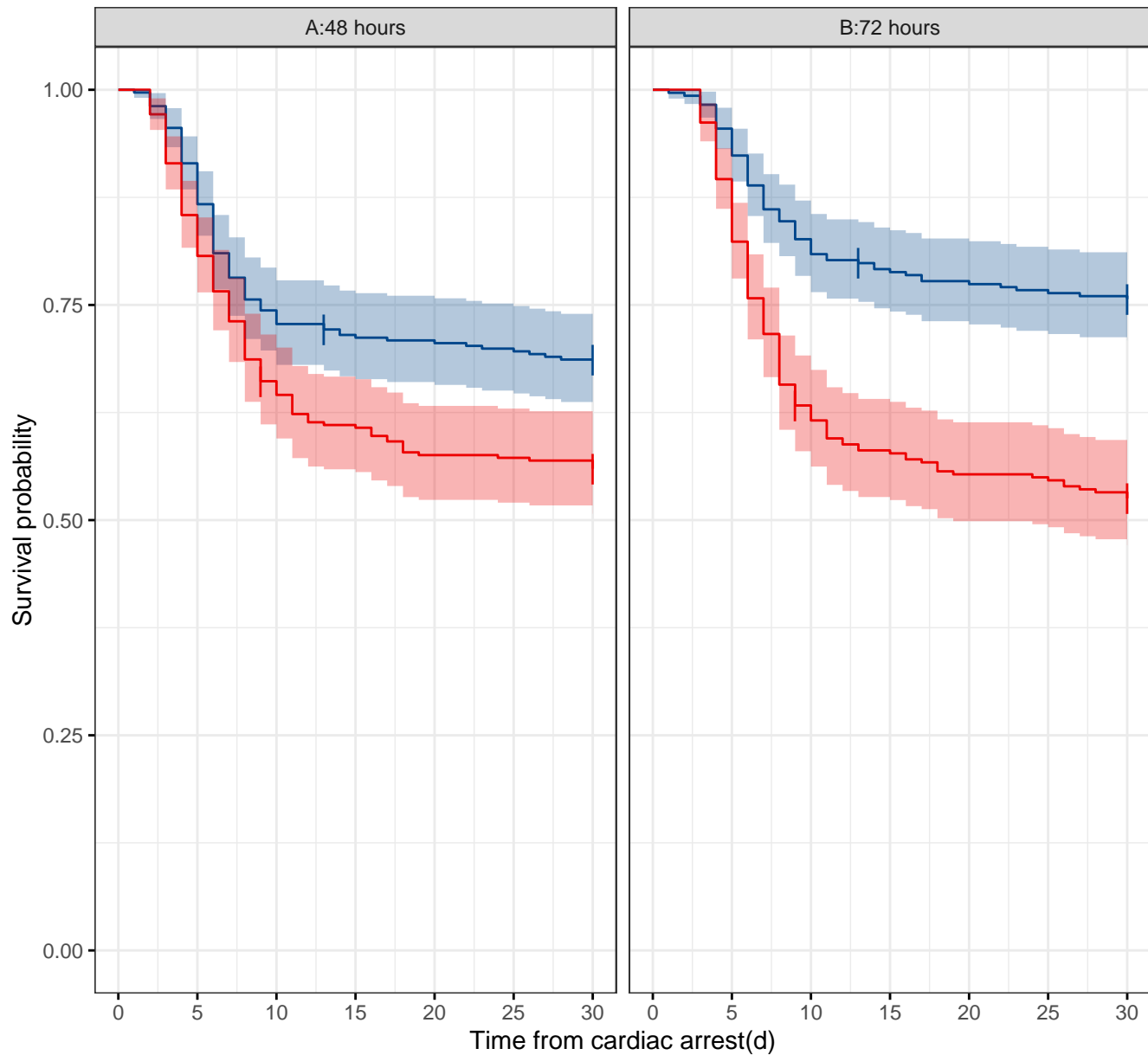

Supplement: Supplementary file 3 — Additional file 3. Kaplan-Meier plots according to copeptin levels stratified as above or below median at 48, and 72 h. Shaded areas indicate 95% confidence interval. Outcome was censored after 30 days. Survival was significantly higher in the group with copeptin levels below median at 48 h, p = 0.001 and 72 h, p < 0.001. [file 13054_2020_2904_MOESM3_ESM.pdf]

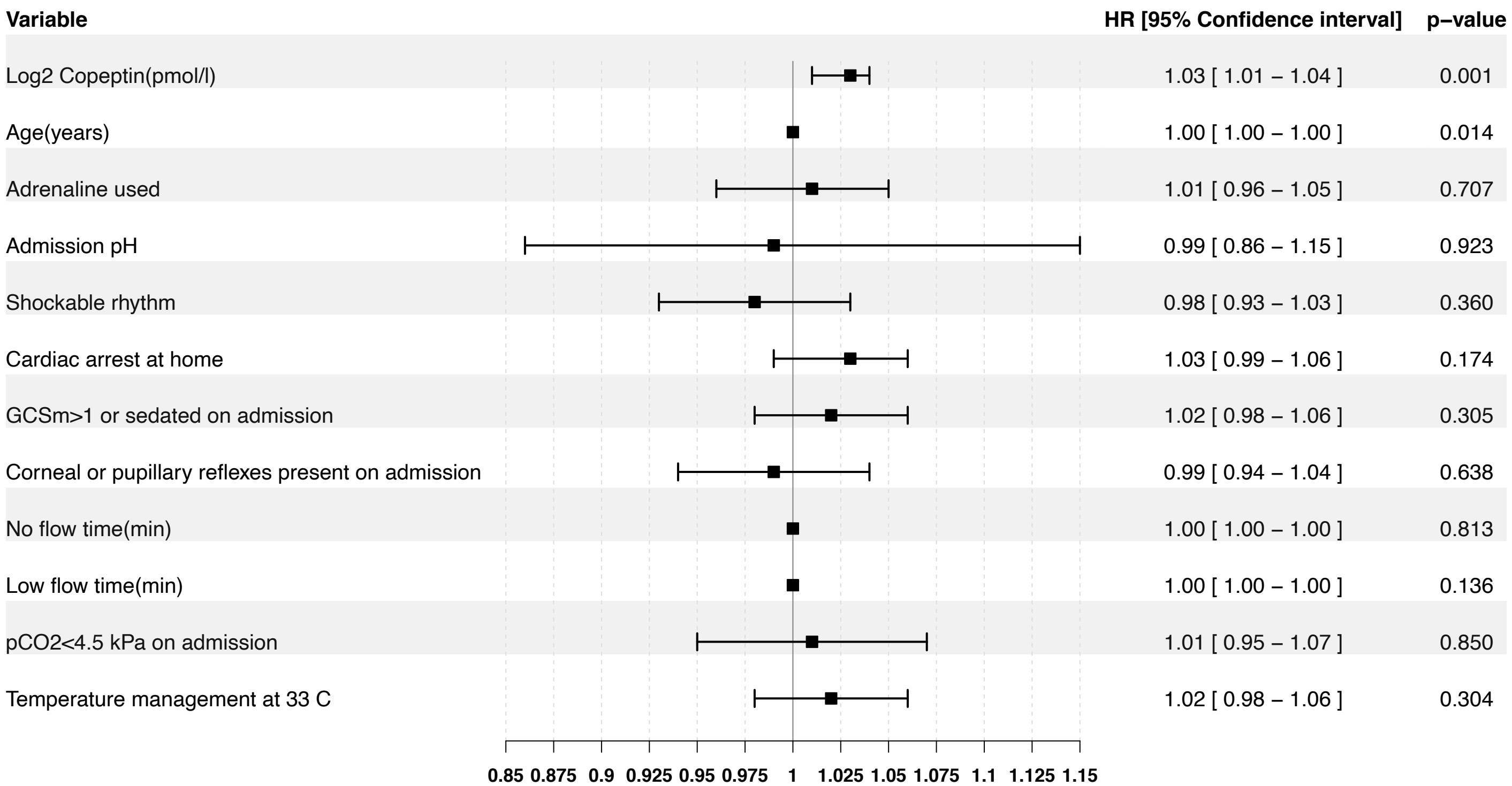

Supplement: Supplementary file 4 — Additional file 4. Forest plot displaying odds ratios for cardiac cause of death within 30 days of cardiac arrest in a multivariate logistic regression mode. The model is adjusted for adjusted for: log2 transformed copeptin at 24 h, age (years), adrenaline used (yes/no), admission pH, Shockable rhythm (yes/no), cardiac arrest at home (yes/no), Glasgow Coma Scale motor component (GCSm) more than 1 or sedated at admission (yes/no), corneal or pupillary reflexes present at admission (yes/no), no flow time = time from cardiac arrest until start of chest compression or return of spontaneous circulation, whichever comes first (min), low flow time = from start of chest compressions until return of spontaneous circulation (min), admission arterial pCO2 below 4.5 kPa on admission (yes/no), and temperature management at 33 C after cardiac arrest (yes/no). p-values below 0.05 were considered significant. [file 13054_2020_2904_MOESM4_ESM.pdf]
